# Role of POCUS in Assessing an Acute Aortic Thrombus

**DOI:** 10.24908/pocus.v8i2.16480

**Published:** 2023-11-27

**Authors:** Zachary Boivin, Emily Mensel, Trent She

**Affiliations:** 1 Emergency Medicine Residency, University of Connecticut Farmington, CT USA; 2 Department of Emergency Medicine, Hartford Hospital Hartford, CT USA

**Keywords:** Aortic, bedside ultrasonography, POCUS, thrombus Presentation

## Abstract

A 67-year-old female patient presented with abdominal pain with a recent diagnosis of paroxysmal atrial fibrillation. Computed tomography (CT) of the abdomen demonstrated a filling defect concerning for an aortic thrombus. Point of care ultrasound (POCUS) confirmed a mobile thrombus in the proximal abdominal aorta in close proximity to several major arterial branches, leading to urgent surgical consultation due to a concern for mesenteric and end-organ ischemia. POCUS played a role in determining patient management in this novel case, and the patient was anticoagulated and ultimately discharged from the hospital.

## Presentation

A 67-year-old female patient with a past medical history of hypertension presented to the emergency department (ED) with abdominal pain. She reported intermittent palpitations for the past three months, fevers for one week, and a recent admission three days prior for a pleural effusion and atrial fibrillation. Her pleural effusion was drained; cytology was negative for malignancy. She was not discharged on rate or rhythm controlling agents, nor was she anticoagulated. She was given urgent outpatient follow up but returned to the ED sooner than her appointment due to worsening symptoms.-

**Figure 1:**
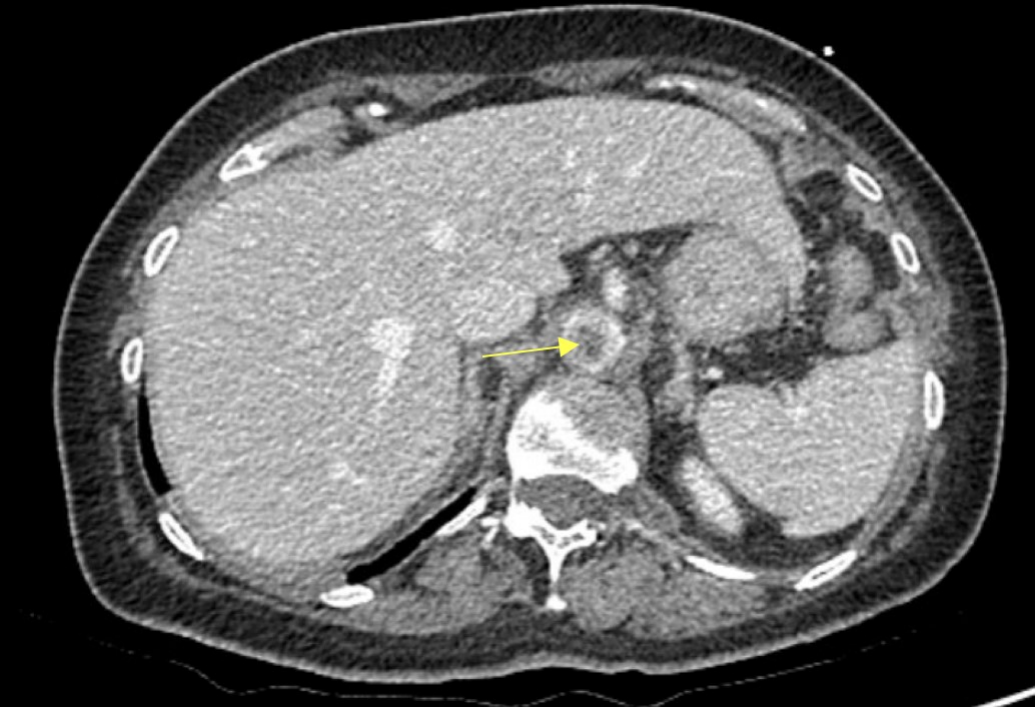
Computed tomography image showing an abdominal aortic thrombus (yellow arrow).

**Figure 2:**
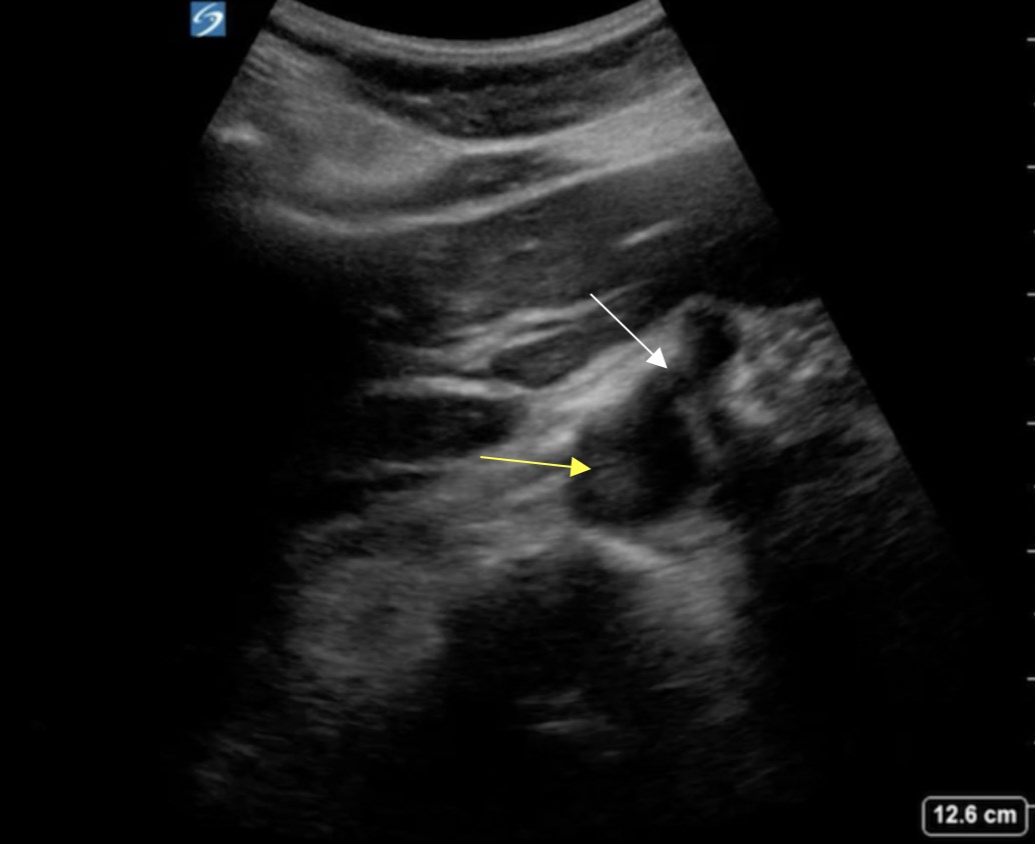
Abdominal aortic thrombus (yellow arrow) at the level of the celiac trunk (white arrow).

In her second ED visit, she complained of upper abdominal pain along with palpitations and weakness. Her physical exam was normal, with her abdomen soft and nontender. She was found to have a leukocytosis of 23.7 x 10^3^/µL, a normal creatinine of 0.8 mg/dL, a rising lactic acid from 1.5 mmol/L to 2.7 mmol/L. A computed tomography (CT) of the abdomen and pelvis with intravenous contrast revealed concern for a proximal aortic thrombus and an acute renal infarction (Figure 1). The patient was placed on a heparin infusion and surgical consultation was obtained. At this time, point of care ultrasound (POCUS) was performed to further assess and characterize the potential thrombus. A mobile, hyperechoic thrombus was located within the aorta at the level of the celiac trunk that moved both proximally and distally with aortic pulsations (Figure 2, Video S1, Video S2). The aorta was not noted to be aneurysmal, nor was a dissection identified on either CT or POCUS. 

After discussion with the ED and vascular surgical team, the patient was deemed to be a high risk for mesenteric ischemia due to thrombus mobility, location, and the presence of a renal infarct. Conservative management was favored due to the patient’s recent fevers and leukocytosis. The patient was admitted to the medical step-down unit with repeat imaging planned in 48 hours with close monitoring to ensure no further signs of thrombus progression. She had a formal transthoracic echocardiogram performed which did not reveal any cardiac abnormalities or shunt. The patient remained on anticoagulation, and she was discharged home after an uneventful seven-day hospital stay with outpatient follow-up. Workups for both hypercoagulability and malignancy were unrevealing. There was concern the aortic thrombus was related to her new-onset atrial fibrillation, but no definitive cause was established.

## Discussion

Previous literature suggests multiple potential causes of aortic thrombi, including intrinsic aortic pathology such as dissection or abdominal aortic aneurysm (AAA), or extrinsic causes such as hypercoagulability 1, 2, 3, 4, 5. Both the aortic thrombus and associated intrinsic aortic pathology can be visualized with POCUS. When an aortic thrombus is associated with a dissection, the echogenic thrombus is seen with a visible flap on POCUS that is thinner and attached to the wall of the aorta 2. An associated thrombus with an AAA (when the aorta is ≥ 3 cm in diameter) is seen in a false lumen surrounding the true lumen of the aorta 6. While an aortic thrombus in the descending aorta can be easily visualized with abdominal POCUS, an aortic arch thrombus can only be visualized with a suprasternal cardiac POCUS view, which can be difficult to obtain 7. A thrombus from atrial fibrillation is typically smaller than from other causes, as it typically breaks off from a larger left atrial thrombus, and can travel distally causing solid organ damage, mesenteric ischemia, or limb ischemia 8, 9. This is the primary reason patients with atrial fibrillation are considered for anticoagulation. We could not find any prior reports of POCUS assessment of an aortic thrombus in the proximal abdominal aorta, as other reports detail use of CT and cardiology-performed echocardiogram 3, 10, 11, 12.

We suspect that this patient was in paroxysmal atrial fibrillation during the three months she reported palpitations, which may have been the cause of her aortic thrombus. She had a calculated CHA2DS2-VASc score of 3 for age, sex, and hypertension, making her a moderate-high risk with a recommendation favoring anticoagulation 13. Without anticoagulation, she was at high risk for both development and propagation of thrombus. While atrial fibrillation offers a potential etiology, especially since no alternate etiology was found, this is an unusual cause of proximal aortic thrombus as thrombi from atrial fibrillation often travel more distally due to its size and the aorta’s high velocity flow 5.

The initial treatment for acute abdominal aortic thrombus is therapeutic anticoagulation with operative intervention considered on a case-by-case basis 4, 11, 14, 15, 16, 17. The selection of therapy must balance the benefit of preventing further embolic complications against the risk of iatrogenic thrombus propagation. Mobile thrombi that are in proximity to solid organs or vascular branches of the aorta, particularly those that are persistent through anticoagulation, often require endovascular or open intervention. POCUS can play a large role in decision making after diagnosis, providing real-time information on thrombus mobility and treatment efficacy during hospitalization, and may decrease the number of repeat CT scans needed to assess for thrombus resolution, saving the patient radiation, time, and contrast loads. This novel use of aortic POCUS is not meant to replace the use of CT, but it can augment patient care during admission as an adjunctive imaging technique with serial POCUS exams to provide additional information about the thrombus. In our case, the mobility of the thrombus as well as its location close to several major aortic vascular branches were key pieces of information ascertained by POCUS that were major factors in our patient’s care. 

## Conclusion

The use of POCUS during this case was important to further characterize the dynamic movement of the thrombus that was not captured on CT scan. Although the information gained from POCUS was utilized for initial decision making, the patient was ultimately discharged on anticoagulation without intervention, with atrial fibrillation as a potential cause of her aortic thrombus given a lack of other identifiable etiology. 

## Statement of Consent

Informed consent was obtained from the patient by the authors. The patient has consented to the use of de-identified images, video clips, and health information to be published within the journal.

## Disclosures

The authors have no disclosures related to this work. 

## Supplementary Material

 Video S1 Abdominal aortic thrombus at the level of the celiac trunk.

Video S2 Fanning through the abdominal aortic thrombus in transverse view.

## References

[R214372329527512] Yee A M, Etebari C V, Adhikari S, Amini R (2017). Point of Care Ultrasound Diagnosis of a Massive Thoracoabdominal Aortic Aneurysm. Cureus.

[R214372329527516] Earl-Royal E, Nguyen P D, Alvarez A, Gharahbaghian L (2019). Detection of Type B Aortic Dissection in the Emergency Department with Point-of-Care Ultrasound. Clin Pract Cases Emerg Med.

[R214372329527519] Khine K, Toor A, Khalighi K, Krishnamurthy M (2019). Incidental descending thoracic aortic thrombus: the conundrum of medical versus surgical therapy. J Community Hosp Intern Med Perspect.

[R214372329527522] Maloberti A, Oliva F, De Chiara B, Giannattasio C (2016). Asymptomatic aortic mural thrombus in a minimally atherosclerotic vessel. Interact Cardiovasc Thorac Surg.

[R214372329527525] Agarwal K K, Douedi S, Alshami A, Dejene B, Kayser R G (2020). Peripheral Embolization of Left Ventricular Thrombus Leading to Acute Bilateral Critical Limb Ischemia: A Rare Phenomenon. Cardiol Res.

[R214372329527513] Beales L, Wolstenhulme S, Evans J A, West R, Scott D J (2011). Reproducibility of ultrasound measurement of the abdominal aorta. Br J Surg.

[R214372329527528] Kinnaman K A, Kimberly H H, Pivetta E, Platz E, Chudgar A, Adduci A (2016). Evaluation of the Aortic Arch from the Suprasternal Notch View Using Focused Cardiac Ultrasound. J Emerg Med.

[R214372329527526] Lyaker M R, Tulman D B, Dimitrova G T, Pin R H, Papadimos T J (2013). Arterial embolism. Int J Crit Illn Inj Sci.

[R214372329527524] Bloom B, Gibbons R, Brandis D, Costantino T G (2020). Point-of-care Ultrasound Diagnosis of Acute Abdominal Aortic Occlusion. Clin Pract Cases Emerg Med.

[R214372329527517] Sugiura T, Dohi Y, Yamashita S, Murai S, Ohte N (2016). A case report of asymptomatic aortic thrombosis incidentally detected by computed tomography in apparently healthy subject with a history of cancer surgery. Thromb J.

[R214372329527521] Yang S, Yu J, Zeng W, Yang L, Teng L, Cui Y (2017). Aortic floating thrombus detected by computed tomography angiography incidentally: Five cases and a literature review. J Thorac Cardiovasc Surg.

[R214372329527527] Quach N, Medina M, Burton É (2021). Incidentally Found Ascending Aortic Thrombus. JACC: Case Reports.

[R214372329527518] Harb S C, Wang Tkm, Nemer D, Wu Y, Cho L, Menon V (2021). )DS(2)-VASc score stratifies mortality risk in patients with and without atrial fibrillation. Open Heart.

[R214372329527514] Martins Apd, Bertolucci L H, Warpechowski R B, Angonese A, De Azevedo M S, Rodrigues C (2022). Mobile thrombus of the abdominal aorta: a narrative review. J Vasc Bras.

[R214372329527520] Meyermann K, Trani J, Caputo F J, Lombardi J V (2017). Descending thoracic aortic mural thrombus presentation and treatment strategies. J Vasc Surg.

[R214372329527515] Fayad Z Y, Semaan E, Fahoum B, Briggs M, Tortolani A, D'Ayala M (2013). Aortic mural thrombus in the normal or minimally atherosclerotic aorta. Ann Vasc Surg.

[R214372329527523] Siani A, Accrocca F, De Vivo G, Mounayergi F, Marcucci G (2016). Endovascular Treatment of Symptomatic Thrombus of the Descending Thoracic Aorta. Ann Vasc Surg.

